# Comprehensive analysis of differences in cashmere production performance in Liaoning cashmere goats by using metabolomics and transcriptomics

**DOI:** 10.3389/fvets.2025.1701332

**Published:** 2025-12-10

**Authors:** Bing Luo, Meiqi Wang, Di Han, Chunqiang Wang, Wei Ma

**Affiliations:** 1 College of Animal Science and Veterinary Medicine, Jinzhou Medical University, Jinzhou, China; 2 Liaoning Province Agriculture and Rural Development Service Center, Shenyang, China

**Keywords:** Liaoning cashmere goat, cashmere production performance, transcriptomics, metabolomics, lipid metabolism

## Abstract

**Introduction:**

The Liaoning cashmere goat is a precious dual-purpose breed in China. Although cashmere yield has improved through years of selective breeding, there remains potential for further enhancement. A subset of these goats exhibits a perennial long-wool (PT-LCG) trait, characterized by higher cashmere yield and an extended fiber growth cycle, whereas others display a seasonal long-wool (ST-LCG) pattern. The molecular mechanisms underlying these distinct traits are not yet fully understood.

**Methods:**

Skin tissue samples were collected from six female PT-LCG and six ST-LCG goats during the catagen phase (November). Histological structure of secondary hair follicles (SHFs) was examined via H&E staining. Untargeted metabolomic profiling was performed using gas chromatography-mass spectrometry (GC-MS) to identify differentially expressed metabolites (DEMs) and KEGG pathway enrichment. Transcriptome sequencing (RNA-seq) was conducted on the Illumina platform to identify differentially expressed genes (DEGs), followed by Gene Ontology (GO) and KEGG enrichment analyses. Key DEGs were validated by qRT-PCR. Integrated multi-omics analysis was employed to explore metabolite-gene interactions.

**Results:**

Histological observation revealed no significant structural differences in SHFs between the two types during catagen; however, PT-LCG showed higher SHF activity. Metabolomics identified 92 DEMs (67 up- and 25 down-regulated), primarily enriched in lipid metabolism, amino acid metabolism, and energy metabolism pathways. Key metabolites such as 3-hydroxypropionic acid and multiple long-chain fatty acids were significantly up-regulated in PT-LCG. Transcriptomic analysis revealed 145 DEGs (28 up- and 117 down-regulated), significantly enriched in lipid metabolism, extracellular matrix (ECM) organization, and inflammatory pathways including PPAR and IL-17 signaling. Integrated analysis uncovered a synergistic “metabolite–receptor–gene” network, indicating that lipid metabolism, energy supply, and ECM remodeling are crucial for maintaining follicle activity in PT-LCG. qRT-PCR confirmed the reliability of the RNA-seq data.

**Discussion:**

This study reveals the molecular basis for prolonged hair follicle activity in PT-LCG through integrated transcriptomic and metabolomic analysis. PT-LCG likely sustains continuous cashmere growth by enhancing lipid synthesis and storage, remodeling the ECM for structural support, and modulating inflammatory signaling such as the IL-17 pathway to maintain follicular microenvironment homeostasis. These findings provide new insights into the regulation of the hair follicle cycle in mammals and offer valuable candidate targets for molecular breeding aimed at improving cashmere yield and quality.

## 1 Introduction

Known as a “national treasure,” the Liaoning cashmere goat has seen improvements in cashmere yield through years of selective breeding, but it still could be enhanced further. Cashmere goat skin contains two types of hair follicles: primary hair follicles (1), which produce coarse hair, and secondary hair follicles, which determine the yield and quality of cashmere. The hair follicles of cashmere goats, whether during the embryonic or postnatal stages, exhibit self-renewal and cyclical growth, progressing through three phases: anagen (growth phase), catagen (regression phase) and telogen (resting phase) (2). This is a complex physiological and biochemical process influenced by many factors that can alter the hair follicle cycle and, consequently, hair growth (3, 4). Therefore, identifying regulatory factors, related genes and skin metabolites that control the growth and development of secondary hair follicles is crucial for elucidating the regulatory mechanisms of the hair follicle cycle, improving cashmere quality and increasing yield. During the breeding of Liaoning cashmere goats, a subset of goats exhibits a perennial long wool trait (cashmere fibers grow year-round), whereas others display a seasonal long wool trait (cashmere growth occurs from July to February, totalling 8 months). The former is characterized by higher cashmere yield and longer fiber growth cycles, but the mechanisms underlying these traits remain unclear.

The application of novel omics technologies has become increasingly widespread. Genomics, transcriptomics, proteomics and metabolomics have all been employed in animal production. Transcriptomics provides a snapshot of gene transcription related to studied phenotypes, and metabolomics bridges the gap between genotype and phenotype. Liu et al. (5) used the Roche 454 platform to perform deep sequencing, *de novo* assembly and annotation of the Liaoning cashmere goat transcriptome, providing rich data for an enhanced understanding of its transcriptome. Zhang et al. (6) conducted blood metabolomics analyses of Inner Mongolia white cashmere goats under shortened and natural photoperiod conditions, revealing that different metabolites may contribute to the regulation of cashmere growth. Recent studies suggest that integrating transcriptomic datasets with genomic and other omics datasets (systems genomics) can enhance the detection of causal and regulatory factors and molecular pathways underlying complex phenotypes or diseases in animals (7, 8). However, the application of multi-omics approaches in animal production, particularly in cashmere goat breeding, has received less attention.

The hair follicle growth cycle in cashmere goats consists of three phases: anagen, catagen and telogen. This study focuses on the skin of PT-LCG and ST-LCG goats during the catagen phase by using high-throughput transcriptomic and metabolomic sequencing technologies to comprehensively analyse the differences in cashmere production performance in Liaoning cashmere goats. The findings aim to provide insights into the fundamental research on cashmere growth regulation and molecular breeding strategies.

## 2 Materials and methods

### 2.1 Ethical approval

All procedures were conducted in accordance with the guidelines established by the Ministry of Agriculture of the People's Republic of China. Ethical approval for this study was obtained from the Ethics Committee of Jinzhou Medical University.

### 2.2 Experimental animals and sample collection

The cashmere goats used in this study were from the PT-LCG and ST-LCG populations at the Liaoning Modern Agricultural Production Base Construction Engineering Center. All goats were raised under identical feeding and environmental conditions.

Cashmere Sample Collection: A 5 × 5 cm^2^ area was selected on the right scapula of each cashmere goat. After measuring the cashmere length with a ruler, the cashmere in this area was clipped close to the skin using curved scissors. The cashmere was then stored in its natural state in sealed bags and labeled for the determination of cashmere fiber diameter (100 samples were collected from each population, all from female cashmere goats).

Skin Sample Collection: During the catagen (November), six PT-LCG and six ST-LCG female cashmere goats (1.5 years old) were selected. Skin samples were collected from the scapula region using a biopsy punch (1 cm in diameter). Each sample was divided into two portions: one was preserved in liquid nitrogen for total RNA analysis, and the other was fixed in 4% paraformaldehyde for H&E staining.

### 2.3 H&E staining of skin tissues

Fixed skin tissues were dehydrated, paraffin-embedded and sectioned into 5 μm slices by using a rotary microtome. The sections were stained following the standard H&E protocols.

### 2.4 Metabolomics analysis

Pre-cooled samples were mixed and vortexed for 30 s, homogenized with a ball mill at 40 Hz for 4 min and then sonicated in ice water for 5 min (four replicates) before centrifugation. Following evaporation in a vacuum concentrator, methoxyamination hydrochloride and BSTFA were added sequentially for incubation. The samples were gradually cooled to room temperature and added with FAME. Chroma TOF software was utilized to extract peaks and perform baseline correction, deconvolution and other analyses of mass spectrometry data. The LECO-FiehnRtx5 database was employed for qualitative analysis on substances, including mass spectral matching and exponential matching of retention time. In accordance with the characteristics of mass spectrometry and the requirements of the results, appropriate methods and parameters were selected to complete the sample preparation. Ultimately, the peaks in QC samples with a detection rate of < 50% or an RSD of over 30% were removed (9). Differentially expressed metabolites (DEMs) were screened against calibrated and quality-controlled metabolite data, and pathways for DEM enrichment were determined using tools in the KEGG database.

### 2.5 Transcriptomics analysis

Total RNA was extracted from tissue samples by using Trizol reagent. RNA purity was measured by a Nanodrop 2000 spectrophotometer, RNA concentration was accurately quantified with Qubit (version 2.0) and RNA integrity was detected with an Agilent 2100 bioanalyzer. The constructed libraries were evaluated and tested on an Agilent Bioanalyzer 2100 System, and then double-ended (PE) sequencing was conducted using the Illumina high-throughput sequencing platform (HiSeq/MiSeq). Raw data were QATTED and evaluated using FASTP software. The filtered reads were aligned to the reference genome by using Hisat2 (10, 11). Fold change (FC) > 1 and FDR ≤ 0.05 were used as screening criteria for differentially expressed genes (DEGs). Biological functions and metabolic pathways were determined by Gene Ontology (GO, http://geneontology.org/) and Kyoto Encyclopedia of Genes and Genomes (KEGG; http://www.genome.jp/kegg/) enrichment analyses.

### 2.6 qRT-PCR validation

The reliability of RNA-seq results was verified by qRT-PCR. Nine candidate genes were randomly selected for qRT-PCR validation. After the total RNA was extracted, cDNA was synthesized using the Total RNA Reverse Transcriptase Kit (Takara, Dalian, China). Real-time PCR was performed using SYBR premixed Ex TaqTM II (Takara) on an ABI 7500 Fast Real-Time PCR System (Applied Biosystems, Foster City, CA, USA). The optimized cycling conditions were denaturation at 94 °C for 5 min, followed by 45 cycles at 94 °C for 15 s and 55 °C for 15 s. Three replicate experiments were performed for each sample. β-actin was used as an internal control for result normalization, and its relative expression was determined using the 2^−ΔΔ*CT*^ method. The primer sequences are shown in Table 1.

**Table 1:** Primer sequences for differential gene verification.

**Gene**	**Primer information (5^′^ → 3^′^)**	**Fragment length**	**Reference gene sequence**
*PM20D1*	F′: AAGATTTGCCCCTTGGGGAG	202	XM_005690403.3
	R′: CTGGAGTTTGGACCTGGGAT		
*PLIN4*	F′: TGCTGCCTTCAGACAAGATGA	162	XM_018050877.1
	R′: TGACTGTCAGCGTGGATTGG		
*MOGAT1*	F′: GCTTTGACCCATGGTGCTTAT	150	XM_005676609.3
	R′: GTGGAACAGTGGCAAAGCAA		
*ACSM1*	F′: TGCCAGGACTGCTCCACTA	222	XM_005697556.3
	R′: TCTCTTGCCCTCCTTCTCCA		
*LNPEP*	F′: CCTCTGAGCAAGGGATGAAGAA	99	XM_018050013.1
	R′: AGCTGAAGCCGATCTTTGGGA		
*TMEM91*	F′: CTGTGAGTGCAGGACTAGGG	207	XM_018063274.1
	R′: AAGCCTTGTTGGTCTTCTGGG		
*RBM47*	F′: GCCTACAAACAGGCTTAGCG	234	XM_005681538.3
	R′: CTGTTACCCGAACTCTGAGGC		
*LCN2*	F′: CCAGTGAGCCTGCACCTTTG	154	XM_018055847.1
	R′: CGGGGCACGTGTTTATTTAGC		
*CA4*	F′: CTATAAAACCCGGGTCGGCA	151	XM_018064150.1
	R′: ACCAGTGCGACGCTGC		

This table lists the primer information for the nine differentially expressed genes used for qRT-PCR validation. F′ denotes the forward primer sequence (5′ → 3′), and R′ denotes the reverse primer sequence (5′ → 3′). The Fragment length is given in base pairs (bp). Reference gene sequence accession numbers are sourced from the NCBI database.

### 2.7 Statistical analysis

Using Image-Pro Plus (version 6.0) software, the secondary hair follicle density and hair follicle ratio of the cross-sectional sections were determined. The secondary hair follicle activity was calculated using the following formula: (hair follicle activity (%) = number of hair follicles with red root sheath/total number of hair follicles × 100%). The values shown were replicated in three biological replicates. SPSS (version 22.0) software was used for statistical analysis of variance, followed by Dunnett multiple comparison test. *P* < 0.05 was considered statistically significant.

## 3 Results

### 3.1 Determination of cashmere phenotype and histological observation of skin follicles

The cashmere samples were tested, cleaned, and processed after being dried naturally. An N-107CCD Wool Fineness Tester (Ningbo Yongxin Optical Co., Ltd., Ningbo, China) was used to determine the diameters of the cashmere fibers. In this measurement, the PT-LCG group exhibited greater cashmere length and finer cashmere fiber diameter compared to the ST-LCG group. However, the differences between the groups were not statistically significant (*P* > 0.05; Table 2).

**Table 2:** Cashmere traits of the two types of cashmere goats.

**Type**	**Number**	**Cashmere length (cm)**	**Cashmere fiber diameter (μm)**
PT-LCG	100	8.92 ± 0.34	15.47 ± 0.91
ST-LCG	100	8.63 ± 0.52	15.93 ± 0.66

This table presents the cashmere length and fiber diameter measurements for Perennial long wool type (PT-LCG) and Seasonal long wool type (ST-LCG) Liaoning cashmere goats. Data are shown as mean ± standard deviation, with a sample size of n = 100 per group. Statistical analysis indicated no significant differences in either cashmere length or fiber diameter between the two groups (*P* > 0.05).

**Figure 1:**
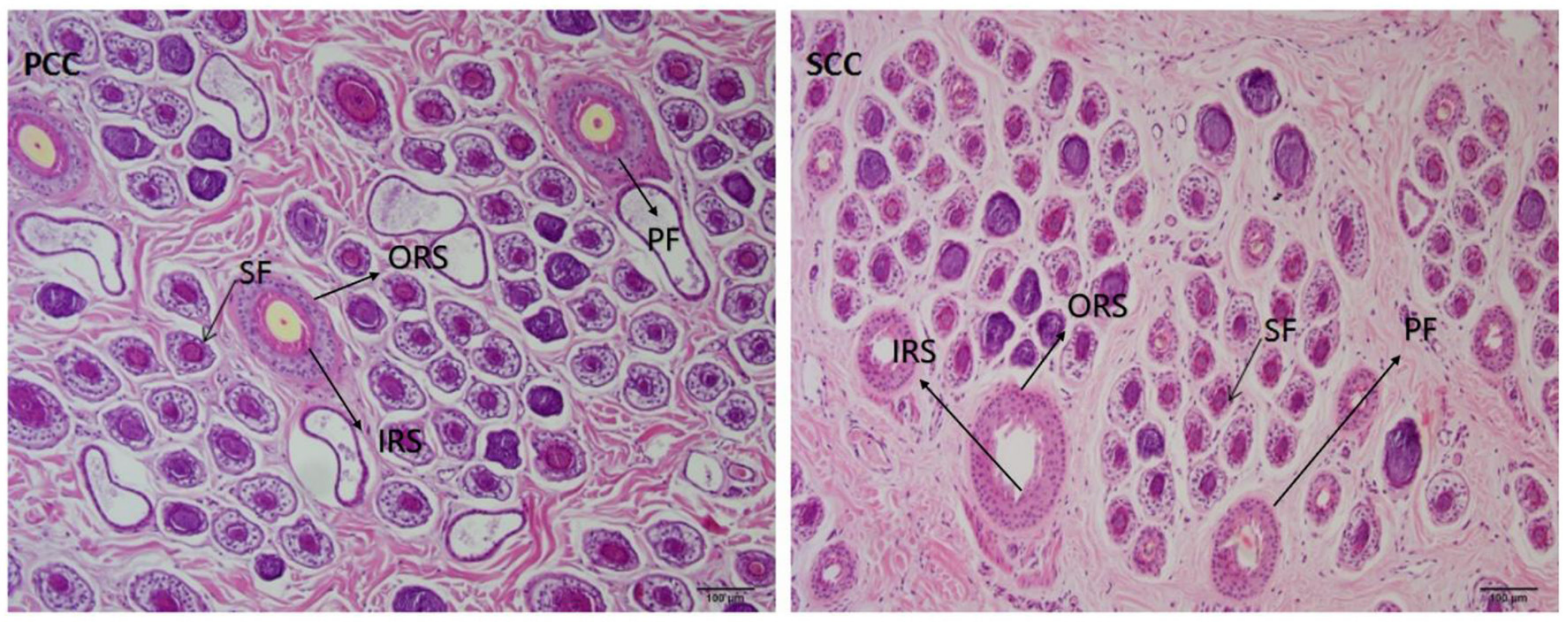
Histological sections of skin hair follicles from two types of cashmere goats during the catagen phase. PF, primary follicle; SF, secondary follicle; ORS, outer root sheath cells; IRS, inner root sheath cells.

H&E staining was used to examine the histological differences between the two types of cashmere during the growth and degeneration phases. As shown in Figure 1, during the catagen, the secondary hair follicles of the two types of cashmere goats have a complete hair follicle structure. The secondary hair follicles are composed of concentric cylindrical structures, from the outside to the inside, as follows: the hoof tissue sheath, the outer heel sheath, the inner heel sheath and the cortex. No difference was observed between the two in terms of morphological composition. In the catagen, the activity of PT-LCG secondary hair follicles decreased significantly. The average secondary hair follicle densities of ST-LCG were 39.03 and 38.24/mm^2^, respectively, with no significant difference. The average secondary hair follicle activities were 91.64 and 90.78%, respectively, without significant difference. The hair follicle ratios were 18.32 and 17.07, respectively, without significant difference (Table 3).

**Table 3:** Analysis of secondary hair follicle characteristics of two types of cashmere goats.

**Type**	**Number**	**Density (number/mm^2^)**	**Activity (%)**	**S/P**
PT-LCG	6	39.03 ± 3.21	91.64 ± 4.18	18.32
ST-LCG	6	38.24 ± 4.06	90.78 ± 5.23	17.07

This table compares the secondary hair follicle characteristics of the two types of Liaoning cashmere goats during the catagen phase. Density is expressed as the number per square millimeter (number/mm^2^). Activity (%) was calculated as (number of hair follicles with red root sheath/total number of hair follicles) × 100%. S/P represents the ratio of secondary to primary hair follicles. Data are presented as mean ± standard deviation, with a sample size of n = 6 per group. Statistical analysis showed no significant differences for any of the parameters listed.

### 3.2 Metabolomics analysis

**Figure 2:**
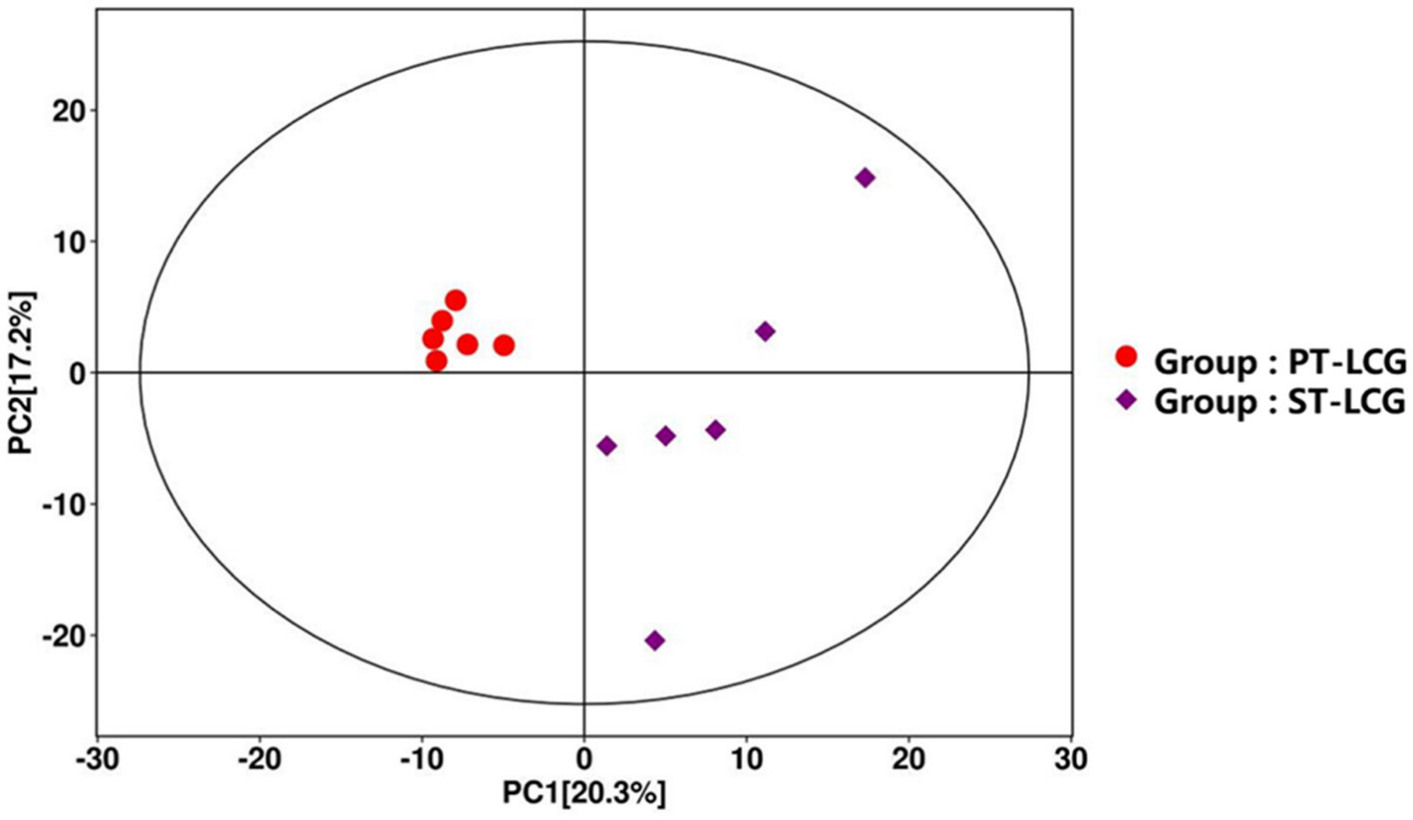
Principal component analysis diagram. Principal component analysis was used to assess the degree of variability between and within the PT-LCG and ST-LCG sample groups. The plot shows a clear separation between the two groups in the principal component space, indicating significant differences in skin tissue metabolites. The red circles represent the PT-LCG group, and the purple squares represent the ST-LCG group.

**Figure 3:**
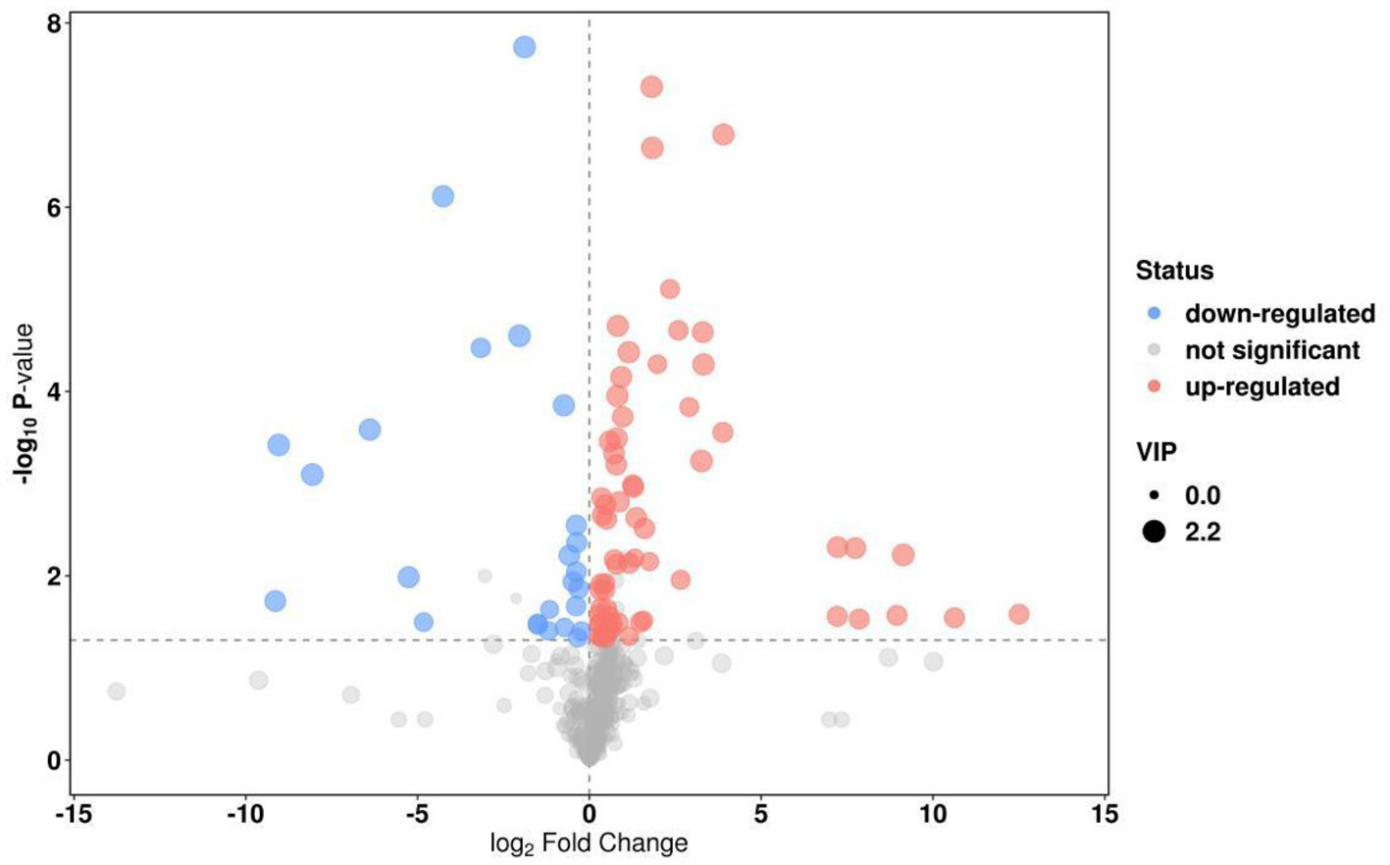
Volcano diagram of differential metabolites. The abscissa represents the fold change, where values increasingly >0.236 indicate a larger magnitude of differential expression; the ordinate represents the level of statistical significance (–log10 transformed *P*-value), where values exceeding 1.3 denote increasingly significant differences. Gray dots indicate non-significant differences; red dots represent significantly upregulated expressions; blue dots represent significantly downregulated expressions.

**Figure 4:**
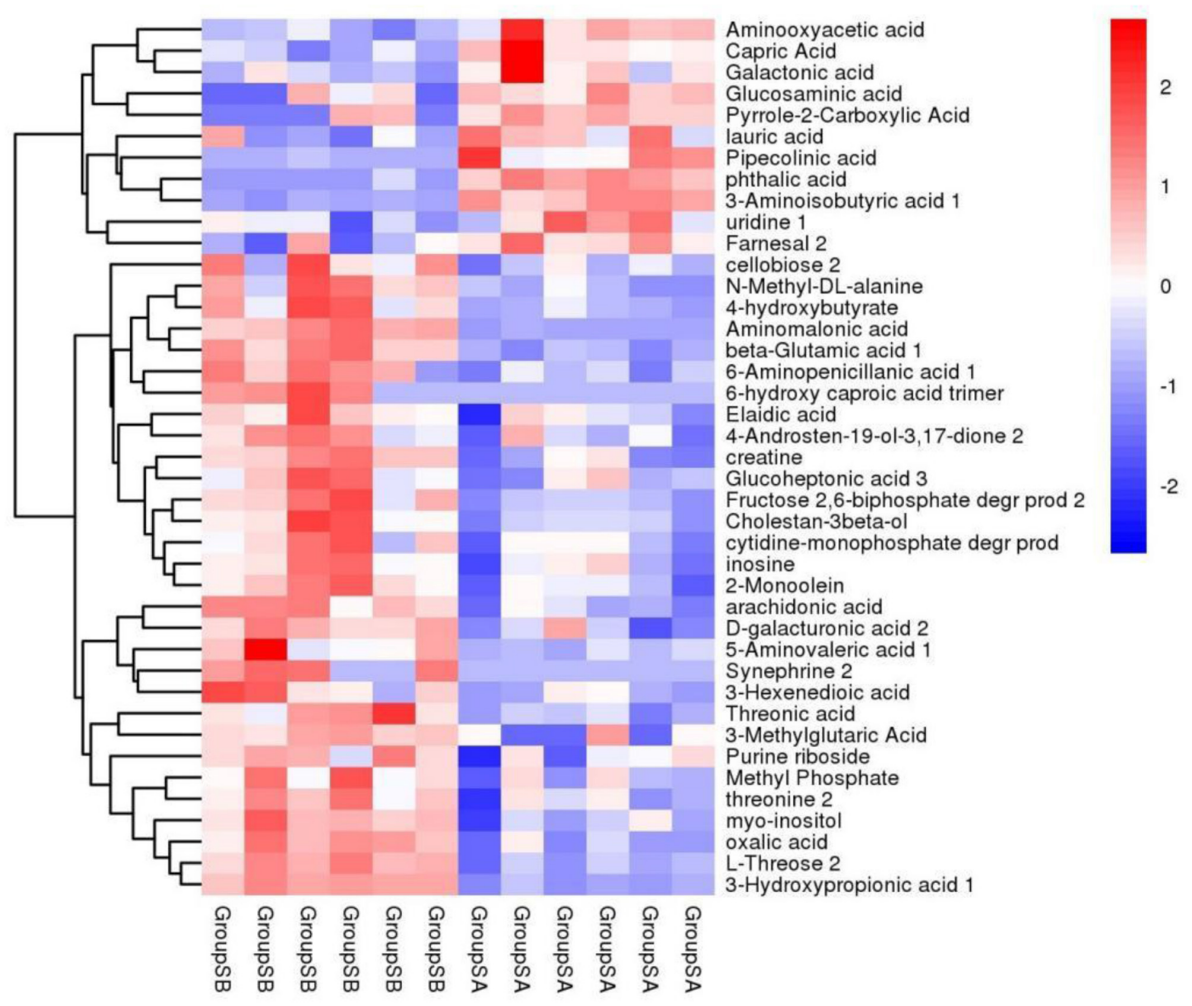
Hierarchical clustering diagram of differential metabolites. The vertical axis represents different clusters, the horizontal axis represents samples from various groups, and the color gradient indicates expression levels, with red denoting high expression and blue denoting low expression.

Principal component analysis (PCA) was employed to evaluate the degree of variability between and within sample groups. As illustrated in Figure 2, significant differences were noted between the two groups. The *P* value of the *t*-test was < 0.05, and the projection importance of the first principal component of the OPLS-DA model was >1. A total of 184 metabolites were produced in the two types of villous skin tissues, of which 92 metabolites exhibited significant changes (67 up-regulated and 25 down-regulated; Figure 3). Some of the screening results are presented in Table 4. A complete list of all differentially expressed metabolites is provided in Supplementary File 1. Differences in the expression patterns of metabolites across different samples are depicted in Figure 4. Forty-six of these differential metabolites were enriched in 11 metabolic pathways according to KEGG (Table 5).

**Table 4:** Differential metabolite screening table (10 cases).

**Id**	**Peak**	**Similarity**	**rt**	**Count**	**VIP**	***P*-value**	**Variation type**
516	Elaidic acid	942	21.1826,0	21	1.299535835	0.047327615	Up
383	Lauric acid	795	15.5186,0	21	1.406713075	0.039890203	Down
566	Inosine	915	24.0557,0	21	1.379914083	0.039164116	Up
551	Cytidine-monophosphate degr prod	809	23.1153,0	21	1.384650255	0.037923859	Up
586	Cellobiose 2	891	25.2595,0	21	1.432601245	0.027386345	Up
292	Capric acid	655	13.1579,0	21	1.667793819	0.011592057	Down
252	Pipecolinic acid	533	11.9178,0	15	1.977987741	0.010342875	Down
344	Threonic acid	881	14.3259,0	21	1.792878751	0.002420103	Up
128	N-Methyl-DL-alanine	811	9.20898,0	21	1.865432126	0.002347726	Up
343	Creatine	895	14.319,0	21	1.766743576	0.001574	Up

This table displays 10 representative differentially expressed metabolites (DEMs) identified through metabolomic analysis. VIP represents the variable importance in the projection. A VIP value > 1.0 was typically used as a screening criterion. In the “variation type” column, “up” indicates up-regulation in the PT-LCG group, and “down” indicates down-regulation.

**Table 5:** Annotation results of differential metabolites KEGG.

**Pathway**	**Description**	**#Compounds (dem)**	**Compounds (dem)**	
			**Metabolites**	**Expression mode**
Chx01100	Metabolic pathways	20	Beta-glutamic acid 1	Up
			Inosine	Up
			Myo-inositol	Up
			Creatine	Up
			Cytidine-monophosphate degr prod	Up
			Lauric acid	Down
			Arachidonic acid	Up
			Oxalic acid	Up
			Capric acid	Down
			Fructose 2,6-biphosphate degr prod 2	Up
			Pipecolinic acid	Down
			4-hydroxybutyrate	Up
			3-hydroxypropionic acid 1	Up
			Phthalic acid	Down
			Galactonic acid	Down
			Threonine 2	Up
			Uridine 1	Down
			D-galacturonic acid 2	Up
			4-Androsten-19-ol-3,17-dione 2	Up
			5-aminovaleric acid 1	Up
			Myo-inositol	Up
			Phthalic acid	Down
			Threonine 2	Up
			Uridine 1	Down
			D-galacturonic acid 2	Up
Chx00240	Pyrimidine metabolism	3	Cytidine-monophosphate degr prod	Up
			3-hydroxypropionic acid 1	Up
			Uridine 1	Down
Chx00330	Arginine and proline metabolism	3	Creatine	Up
			Pyrrole-2-carboxylic acid	Down
			5-aminovaleric acid 1	Up
Chx00052	Galactose metabolism	2	Myo-inositol	Up
			Galactonic acid	Down

This table shows the KEGG pathway enrichment results for the differentially expressed metabolites. The “#compounds (dem)” column indicates the number of DEMs enriched in the corresponding pathway. In the “expression mode” column, “up” signifies up-regulation in the PT-LCG group, and “down” signifies down-regulation.

### 3.3 Transcriptomics analysis

#### 3.3.1 RNA sequence analysis

The six cDNA libraries constructed produced a total of 135,294,737 raw readouts. After low-quality and adapter sequences were filtered out, more than 90% of the remaining valid reads persisted. Amongst the valid reads, the proportion of bases with a mass value of ≥ 20 (sequencing error rate < 0.01) was 99.60%−99.69%, the proportion of bases with a mass value of ≥ 30 (sequencing error rate < 0.001) was 97.05%−97.78%, the GC content was higher than 49.50% and 86.93%−92.09% of the sequences were mapped to the reference genome (Table 6).

**Table 6:** RNA-seq data statistics and summary.

**Sample**	**Raw reads**	**Clean reads**	**Q20 (%)**	**Q30 (%)**	**GC content (%)**	**Total mapped**
A1	22,982,756	21,179,937 (92.16%)	98.76%	95.28%	51.20%	19,337,043 (91.30%)
A2	23,473,385	21,595,562 (92.00%)	98.75%	95.28%	51.04%	19,703,782 (91.24%)
A3	21,627,704	19,911,927 (92.07%)	97.17%	92.71%	51.12%	18,279,828 (91.80%)
B1	20,012,955	18,373,593 (91.81%)	97.06%	92.68%	49.50%	15,972,672 (86.93%)
B2	24,560,483	22,594,769 (92.00%)	98.69%	95.11%	50.75%	20,752,567 (91.85%)
B3	22,637,454	20,887,080 (91.53%)	98.67%	95.05%	51.21%	19,236,328 (92.09%)

This table summarizes the RNA-seq data quality metrics for the six samples. Q20 and Q30 represent the percentages of bases with phred quality scores >20 (sequencing error rate < 1%) and 30 (sequencing error rate < 0.1%), respectively. GC content is the percentage of guanine and cytosine bases among the total bases. “Total mapped” indicates the percentage of reads successfully aligned to the reference genome.

#### 3.3.2 Differentially expressed genes

**Figure 5:**
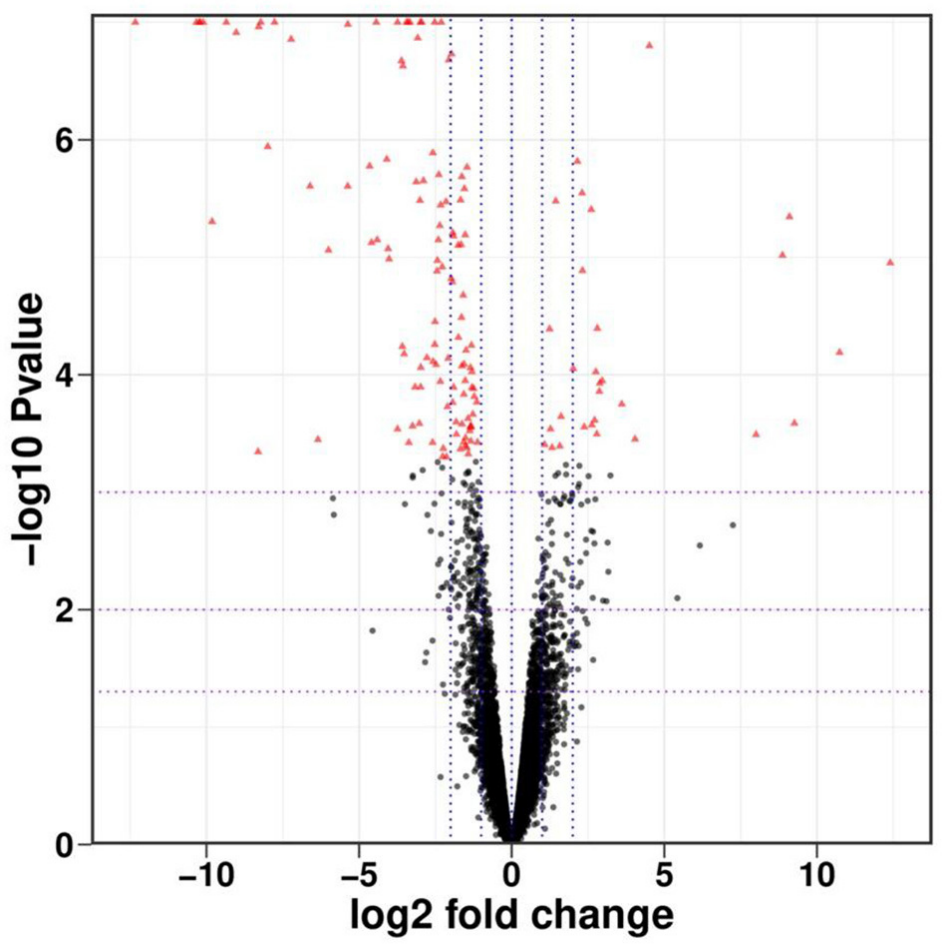
Volcano diagram of differential genes. The *x*-axis represents the log2 fold change in expression, and the *y*-axis represents the –log10 *P*-value. Data points are colored as follows: red for significantly up-regulated, black for significantly down-regulated, and gray for non-significant genes.

**Figure 6:**
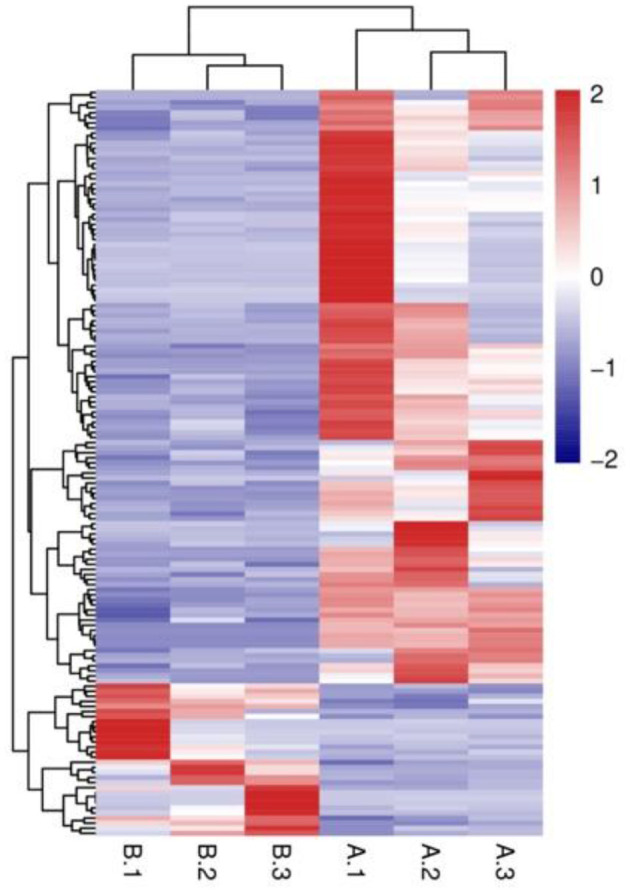
FPKM hierarchical clustering diagram. The vertical axis represents different clusters, the horizontal axis represen ts samples from various groups, and the color gradient indicates expression levels, with red denoting high expression and blue denoting low expression.

The gene expression levels were quantified by FPKM. A total of 145 DEGs, including 28 up-regulated genes and 117 down-regulated genes, were identified in the six cDNA libraries (Figure 5 volcano plot), some of which are shown in Table 7. The FPKM hierarchical clustering heat map (Figure 6) provides a visual representation of the expression patterns of genes in the sample and highlights the reproducibility and confidence of the data.

**Table 7:** Representative differential genes.

**Gene name**	**log2 (fc)**	**qval**	**Regulation**	**Significant**
*PM20D1*	−4.082793565	1.32E-06	Down	Yes
*ACSM1*	−3.66685197	5.66E-05	Down	Yes
*UCN3*	3.036117577	7.21E-07	Up	Yes
*AMH*	2.971033383	9.59E-06	Up	Yes
*SCD*	−2.958394198	1.17E-04	Down	Yes
*PSAPL1*	−2.93481364	1.55E-12	Down	Yes
*LCN2*	1.740741837	4.02E-06	Up	Yes
*MBOAT2*	−1.733282649	1.77E-07	Down	Yes
*KRT9*	1.731413031	6.16E-04	Up	Yes

This table lists some representative differentially expressed genes (DEGs). The ‘log2(fc)' column represents the log2-transformed fold change in gene expression. The “qval” column shows the P-value adjusted for multiple testing. The “regulation” column indicates the direction of expression change in the PT-LCG group (“up” for up-regulated, “down” for down-regulated). The “significant” column denotes whether the differential expression was statistically significant.

#### 3.3.3 Analysis of DEGs

**Figure 7:**
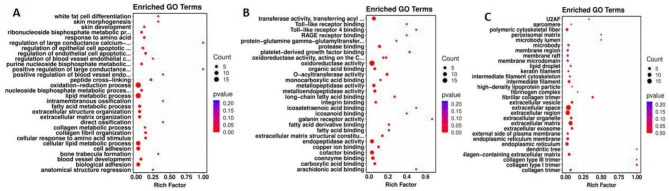
GO enrichment map. The abscissa represents the enrichment score, the ordinate represents the enriched terms. The size of the bubbles corresponds to the number of enriched genes. **(A–C)** represent Biological Process, Molecular Function, and Cellular Component, respectively.

GO enrichment assays were utilized to annotate DEGs and study their distribution to further elucidate the function of DEGs. A specific *P*-value was employed to determine whether the GO function is enriched. In general, a *P* value of < 0.05 indicates enrichment. In the comparison group, the majority of DEGs were involved in biological processes, molecular functions and cellular components. Amongst them, 11 GO entries were significantly enriched in biological processes, predominantly affecting the lipid metabolic process and the regulation of endothelial and epithelial cell apoptotic processes. In terms of molecular functions, 37 GO items were significantly enriched, mainly pertaining to enzyme activity, fatty acid binding and organic acid binding. Regarding cell components, 11 GO entries were significantly enriched, predominantly acting on extracellular matrix (ECM) and extracellular exosomes. The results of GO enrichment analysis are illustrated in Figure 7.

**Figure 8:**
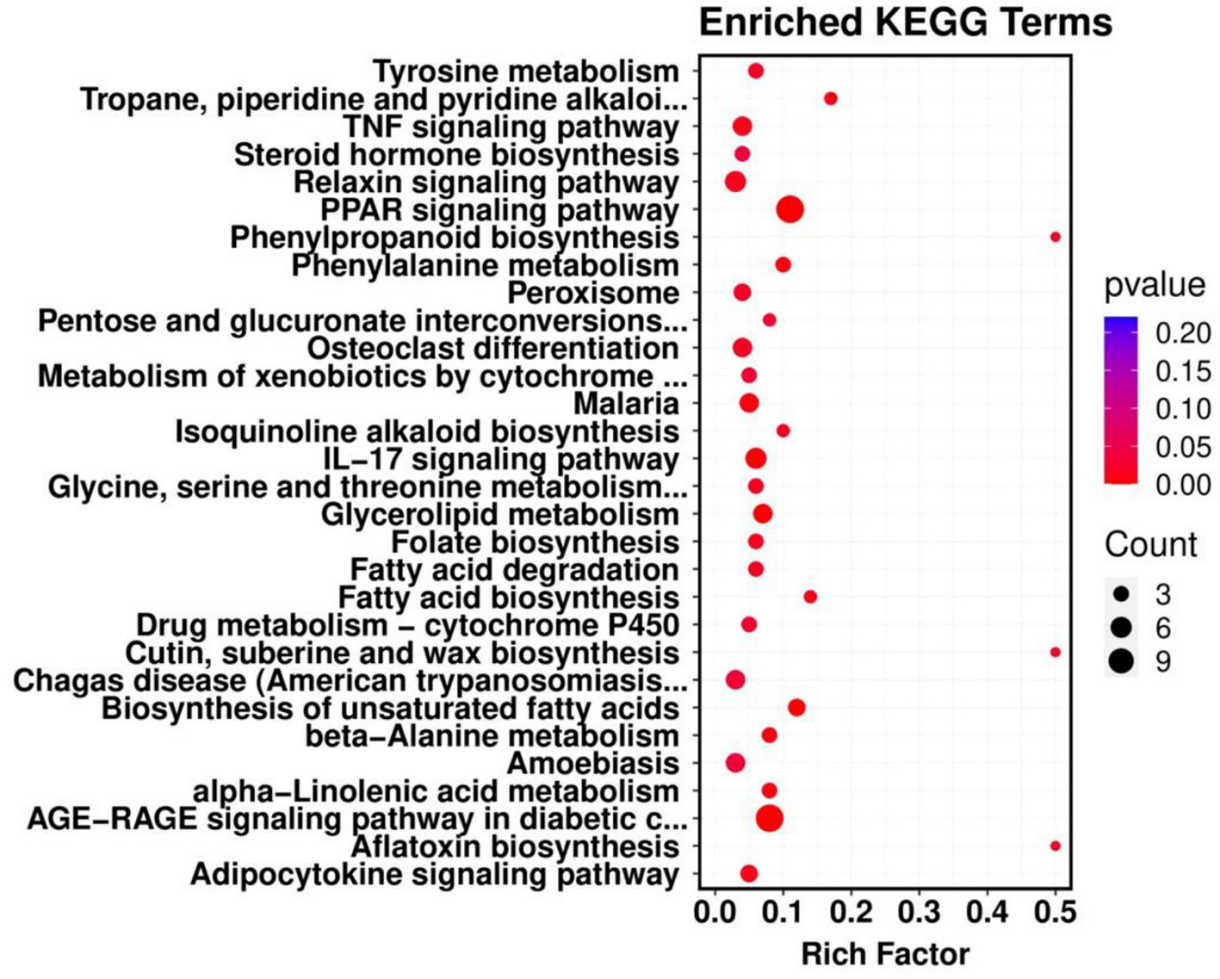
KEGG pathway enrichment map of differentially differentiated genes. The abscissa represents the enrichment score, the ordinate represents the signaling pathways. The bubble size corresponds to the number of enriched genes.

KEGG pathway enrichment analysis was conducted to further identify the major biochemical, metabolic and signal transduction pathways of DEGs. The results showed that a total of 399 genes were mapped to 179 KEGG pathways, of which 37 genes were significantly enriched in five KEGG pathways (*P* < 0.05). These pathways are involved in the PPAR signaling pathway, the AGE-RAGE signaling pathway, the biosynthesis of unsaturated fatty acids, the IL-17 signaling pathway and glycerine metabolism in diabetic complications (Figure 8).

### 3.4 Transcriptome and metabolome correlation analysis

**Figure 9:**
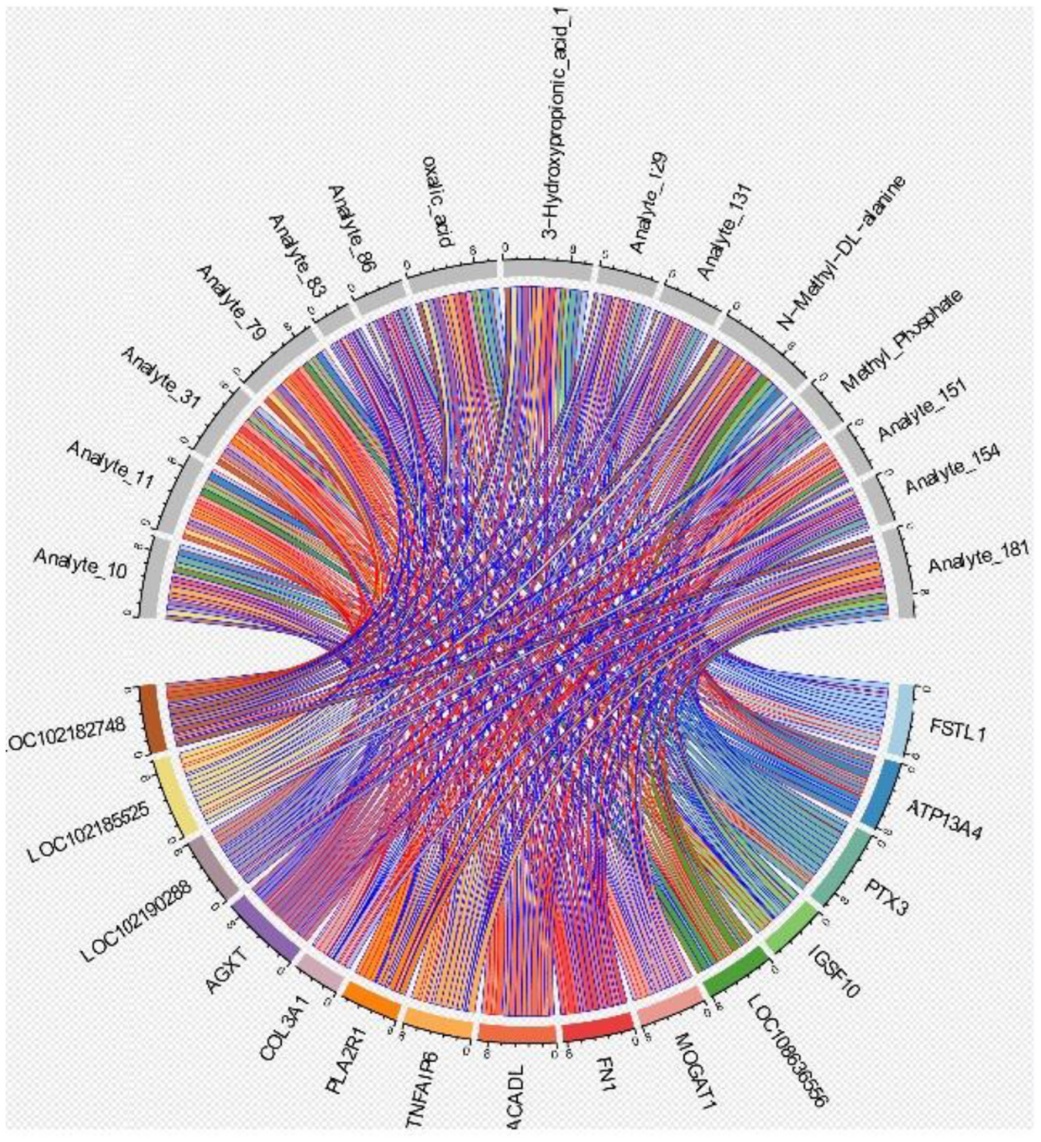
Correlation analysis chart. This chord diagram illustrates the correlations between the top 10 differentially expressed genes and the top 10 differential metabolites. The connecting lines, color-coded by correlation pair, represent statistically significant associations between the corresponding gene and metabolite.

The association results of the top 10 DEGs and the top 10 DEMs were integrated to assess the association between the transcriptome and metabolome (Figure 9). The results showed that the correlation coefficients between the genes ATP13A4, AGXT, ACADL, FN1 and FSTL1 and the metabolite N-methyl-DL-alanine were higher than 0.70, and the correlation coefficients of the genes FSTL1, LOC102190288, MOGAT1, PTX3, TNFAIP6 and AGXT and 3-hydroxypropionic acid_1 were higher than 0.70.

### 3.5 qRT-PCR validation results

**Figure 10:**
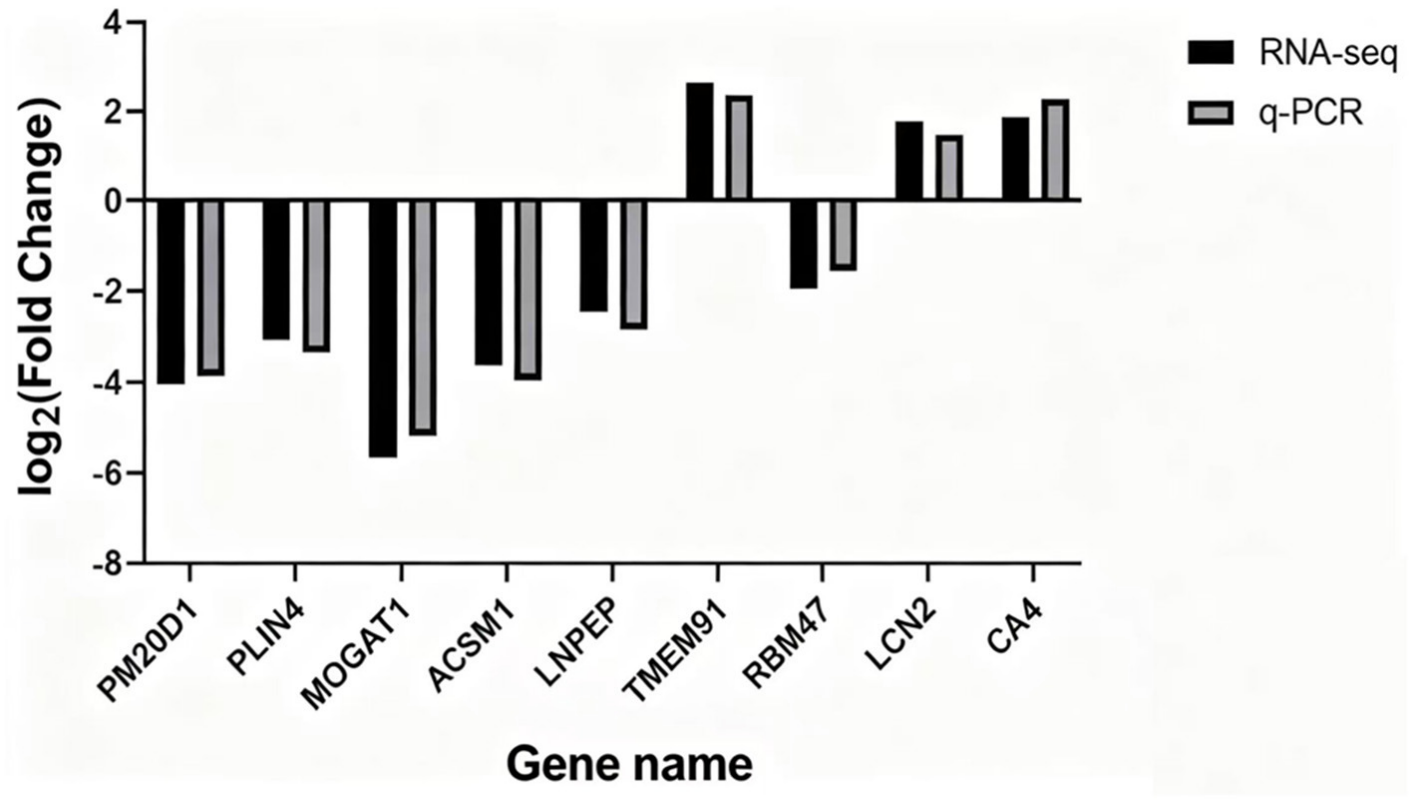
Validation of RNA-seq data by qRT-PCR. Bar graph comparing the log2 fold change (log2FC) of nine differentially expressed genes between RNA-seq (blue bars) and qRT-PCR (red bars) results. The gene names are indicated on the x-axis. The y-axis represents the log2 fold change (log2FC) values.

Nine DEGs were randomly chosen for real-time PCR to validate the transcriptome analysis results of RNA-seq. Amongst them, TMEM91, LCN2 and CA4 were up-regulated, whereas PM20D1, PLIN4, MOGAT1, ACSM1, LNPEP and RBM47 were down-regulated. Compared with the RNA-seq results, the qRT-PCR results of the nine genes indicated that although some differences existed in the expression levels, the difference was not significant (*P* > 0.05), and the expression trend was consistent (Figure 10), indicating that the RNA-seq results were reliable.

## 4 Discussion

### 4.1 Differences in histological features of secondary hair follicles

The development and periodic changes of secondary hair follicles in cashmere goat skin are an extremely complex physiological process, which is affected by several factors of genetics, nutrition and environment. The differences in the development of hair follicles in various animals have different degrees. In this study, the H&E staining results showed no significant difference in the secondary hair follicle structure of the two cashmere goats during the growth phase. However, the secondary hair follicle activity of ST-LCG was significantly reduced in the catagen stage. This finding is basically consistent with the results of Sun Limin and Han et al. (12, 13), who showed that PT-LCG type has a long velvet cycle and a high down yield, but it does not reduce the villus quality. In addition, no significant difference was found in the secondary follicle density and hair follicle ratio, suggesting that the maintenance of hair follicle number may not be the main factor in the difference in hair production, and the prolongation of hair follicle activity and growth cycle (e.g., the perennial velvet characteristics of PT-LCG) may be more critical.

### 4.2 Regulatory mechanisms revealed by metabolomics

The metabolomic analysis identified 184 metabolites in the skin tissues of the two groups of cashmere goats, of which 92 were DEM, with 67 being up-regulated and 25 down-regulated. These metabolites are primarily enriched in lipid metabolism, amino acid metabolism and energy metabolism pathways.

In the lipid metabolism pathway, 3-hydroxypropionic acid and various long-chain fatty acids (such as palmitic acid and oleic acid) were significantly up-regulated in PT-LCG. 3-Hydroxypropionic acid is an intermediate product of lipid metabolism, it and may promote hair follicle growth through two pathways. Firstly, in terms of energy supply, it acts as a precursor of acetyl-CoA, which provides energy to hair follicle cells (14). Secondly, regarding membrane synthesis, unsaturated fatty acids are vital components of cell membranes, and their increase may facilitate the continuous growth of hair follicles by enhancing the membrane fluidity of hair follicle cells (15).

The enrichment of the PPAR signaling pathway further supports the importance of lipid metabolism in the hair follicle cycle. PPARγ is a key regulator of adipocyte differentiation, and its activation may indirectly support nutrient supply to hair follicles by promoting the development of peri-adipose tissue (16).

The expression levels of N-methyl-DL-alanine and glycine were significantly correlated with hair follicle activity. N-methyl-DL-alanine may regulate the self-renewal of hair follicle stem cells by regulating methylation reactions by influencing epigenetic modifications (17). Glycine is an important substrate for collagen synthesis, and metabolic changes may affect the structural integrity of the outer root sheath of the hair follicle (18).

In the energy metabolism pathway, the expression levels of intermediate products of glycolysis and tricarboxylic acid (TCA) cycle (such as citric acid and succinic acid) were up-regulated in PT-LCG, suggesting that the skin tissue of perennial long-cashmere goats may adapt to the continuous growth needs of hair follicles by enhancing energy metabolism. This finding is consistent with that of Zhang et al. (6) on Inner Mongolia white cashmere goats, suggesting that energy metabolism reprogramming is an important regulatory mechanism for villus growth.

### 4.3 Gene regulatory networks revealed by transcriptomic analysis

A total of 145 DEGs were identified by transcriptomic analysis, including 28 up-regulated genes and 117 down-regulated genes. In terms of the regulatory role of lipid metabolism-related genes, the GO enrichment analysis showed that DEGs were significantly enriched in lipid metabolic process, including fatty acid binding and enzyme activity. Amongst them, the expression levels of ACADL (acyl-CoA dehydrogenase long chain) and MOGAT1 (monoacylglycerol acyltransferase 1) were particularly significant. ACADL is involved in the β-oxidation of fatty acids, and its downregulation may reduce lipid breakdown, thereby retaining more energy for hair follicle cells (19). MOGAT1 catalyses glyceride synthesis, and its upregulation may promote lipid storage and provide a sustained energy supply to hair follicles (20).

In terms of the role of ECM and hair follicle microenvironment, ECM-related genes (such as fibronectin 1 [FN1] and FSTL1) were significantly upregulated in PT-LCG. FN1 is a major component of the ECM, and its increased expression may support hair follicle growth in two manners. Firstly, it enhances the mechanical connection between hair follicle and dermis through the integrin signaling pathway, which provides structural support for hair follicle growth (21). Secondly, it can regulate the proliferation and differentiation of hair follicle stem cells by binding to growth factors, such as TGF-β, and regulate hair follicle growth from the aspect of growth factors (22).

In terms of inflammation and immune regulation, the KEGG analysis showed that the IL-17 and AGE-RAGE signaling pathways in diabetic complications were significantly enriched. IL-17 may accelerate hair follicle degeneration by recruiting immune cells and play a certain role in pro-inflammatory processes. In addition, IL-17 can promote the activation of hair follicle stem cells by activating Wnt signaling, which plays a certain role in the regulation of regeneration (23).

### 4.4 Cross-talk among core signaling pathways

Our integrated transcriptomic and metabolomic analysis has highlighted the critical roles of lipid metabolism, ECM remodeling, and the IL-17 inflammatory signaling pathway in maintaining hair follicle activity in PT-LCG. However, these pathways likely do not operate in isolation but rather engage in intricate cross-talk, collectively forming a coordinated regulatory network.

First, a functional interaction may exist between the IL-17 signaling pathway and lipid metabolism pathways, such as the PPAR signaling pathway. Previous studies have indicated that IL-17 can indirectly suppress the transcriptional activity of PPARγ by activating downstream factors like NF-κB, thereby influencing adipocyte differentiation and lipid storage (24). Particularly in the skin and hair follicle microenvironment, the balance between inflammatory signals and metabolic homeostasis is crucial. Research in psoriasis models has demonstrated that IL-17 can directly inhibit PPARγ expression in human keratinocytes, disrupting lipid barrier function (25). In our study, the concurrent enrichment of the PPAR signaling pathway and the IL-17 pathway in PT-LCG suggests a potential balancing mechanism, whereby the pro-inflammatory effects of IL-17, which may promote hair follicle stem cell activation, are buffered by enhanced lipid metabolism to prevent excessive inflammation-induced follicle regression. Future investigations employing Western Blot analysis to examine the phosphorylation and expression levels of key proteins such as IL-17RA and PPARγ could clarify whether a direct regulatory relationship exists between these pathways.

Second, feedback regulation may occur between ECM remodeling and energy metabolism. ECM components such as FN1 not only provide structural support for hair follicles via integrin signaling but may also enhance cellular glucose uptake and utilization by activating the FAK/PI3K/AKT signaling axis, thereby influencing energy metabolism (21, 26). This signal transduction from the extracellular matrix to intracellular energy metabolism is recognized as “mechano-metabolic coupling” (27). In our study, the upregulation of ECM-related genes (e.g., FN1, FSTL1) coincided with the accumulation of TCA cycle intermediates (e.g., citrate, succinate) in PT-LCG skin tissues, suggesting that ECM-integrin signaling may indirectly sustain the high metabolic demands of hair follicles by modulating cellular energy status.

In summary, a complex network likely exists in PT-LCG, with ECM-integrin signaling as the structural foundation, lipid and energy metabolism as the functional support, and pathways like IL-17 serving as regulatory hubs. This multi-layered cross-talk among pathways may represent the key systems-level biological basis enabling PT-LCG to overcome seasonal constraints and maintain prolonged hair follicle activity. Subsequent research incorporating proteomics, phosphoproteomics, and *in vitro* functional assays is warranted to further validate the direct regulatory interactions among these pathways.

### 4.5 Synergistic effects of multi-omics-integrated analysis

Three key regulatory modules were identified through multi-omics integration: (1) lipid metabolism module, which contains genes, such as ACADL and MOGAT1, and metabolites such as 3-hydroxypropionic acid; (2) ECM module, with *FN1* and *FSTL1* as the core; and (3) energy metabolism module, which involves ATP13A4 genes and TCA cycle intermediates.

The multi-omics data collectively depict a state of sustained anabolism in PT-LCG skin. The downregulation of ACADL (fatty acid β-oxidation) alongside the accumulation of long-chain fatty acids and 3-hydroxypropionic acid suggests a metabolic shift from lipid catabolism toward synthesis and storage. This coordinated “gene-metabolite” pattern provides a continuous supply of structural lipids and energy reserves, essential for supporting prolonged follicle activity.

The lipid metabolism module provides membrane phospholipid synthesis raw materials for the ECM module, the energy metabolism module provides ATP support for the other two modules and the ECM module regulates lipid metabolism through integrin signaling feedback (28). This “metabolite-receptor-gene” cascade of regulation explains, to some extent, why PT-LCG can break through seasonal restrictions to maintain hair follicle activity and provide a certain perspective for understanding the regulation of hair cycle in other mammals.

Although the multi-omics integration yielded some findings, it still has limitations. For example, the sample size was relatively small (*n* = 6/group), which may affect the statistical power, and the spatial omics information were insufficient. Longitudinal sampling combined with single-cell sequencing will be applied in the future to improve resolution. Spatial metabolomics analysis and other studies will be conducted to offer insights into enhancing the breeding efficiency of cashmere goats.

### 4.6 Future perspectives for functional validation

While this multi-omics study identified key candidate molecules and pathways, direct functional validation of core hits like 3-hydroxypropionic acid, ACADL, and FN1 is required to establish causality. Our future work will therefore focus on:

1. Metabolite function: treating cashmere goat dermal papilla cells and fibroblasts with 3-hydroxypropionic acid to assess its effects on proliferation, apoptosis, and expression of hair follicle markers (e.g., KRT9).
2. Gene function: employing siRNA-mediated knockdown of ACADL and overexpression of FN1 in relevant cell models to delineate their roles in lipid metabolism, ECM synthesis, and hair follicle activity.
3. Pathway crosstalk: investigating interactions between the identified modules, for example, by examining how FN1 overexpression influences lipid metabolism gene expression.

These planned experiments will directly test the hypotheses generated here, ultimately clarifying the mechanistic basis for sustained follicle activity in PT-LCG and providing robust targets for molecular breeding.

## 5 Conclusion

This study integrated transcriptomic and metabolomic analyses to elucidate the molecular mechanisms underlying the differences in cashmere production performance between perennial long wool type (PT-LCG) and seasonal long wool type (ST-LCG) Liaoning cashmere goats. Our findings demonstrate that PT-LCG maintains prolonged hair follicle activity through a synergistic regulatory network involving enhanced lipid metabolism, extracellular matrix (ECM) remodeling, and modulation of inflammatory signaling pathways such as IL-17. Key metabolites (e.g., 3-hydroxypropionic acid, long-chain fatty acids) and genes (e.g., ACADL, FN1, MOGAT1) were identified as central components of this coordinated network, which collectively supports continuous follicle growth by ensuring energy supply, structural integrity, and a balanced follicular microenvironment. These insights not only advance our understanding of hair follicle cycle regulation in cashmere goats but also provide valuable candidate targets for molecular breeding strategies aimed at concurrently improving cashmere yield and quality.

Notwithstanding these insights, several limitations of the present study warrant consideration. Firstly, the relatively small sample size (*n* = 6 per group) may constrain the statistical power and limit the exploration of intra-group heterogeneity. Secondly, the absence of proteomic data precludes validation of whether the observed transcriptional changes in key differentially expressed genes (e.g., MOGAT1) translate to the protein level. Furthermore, the bulk tissue omics approach lacks the resolution to delineate cell-type-specific contributions (e.g., from dermal papilla cells or hair follicle stem cells) or to ascertain the spatial localization of the identified metabolites.

Building upon these findings and addressing the current limitations, our future research will prioritize the following directions:

1. Cohort expansion: subsequent studies will incorporate a larger cohort to enhance the robustness and generalizability of the findings.
2. Proteomic integration: TMT-based quantitative proteomics will be employed to validate the expression of pivotal target genes at the protein level, thereby clarifying the transcript-translation consistency.
3. Functional validation: *In vitro* functional assays utilizing cashmere goat skin cell cultures (e.g., dermal papilla cells, fibroblasts) will be conducted. These will involve treatment with core metabolites (e.g., 3-hydroxypropionic acid), siRNA-mediated knockdown (e.g., of ACADL), or overexpression (e.g., of FN1) to directly investigate their causal effects on cell proliferation, apoptosis, lipid metabolism, and the expression of hair follicle activity markers.
4. High-resolution profiling: the application of single-cell RNA sequencing and spatial metabolomics will be pursued to precisely resolve the regulatory mechanisms at a cell-type-specific resolution and within their spatial context, complemented by longitudinal sampling to dynamically capture molecular events throughout the hair follicle cycle.

We anticipate that these forthcoming investigations will rigorously test the hypotheses generated herein and ultimately elucidate the precise mechanisms enabling PT-LCG to overcome seasonal constraints, thereby providing a solid foundation for the genetic improvement of cashmere goats.

## Data Availability

The whole-genome sequencing data generated in this study are openly available in the NCBI database. The raw reads are available in the Sequence Read Archive under BioProject accession PRJNA1367900. The metabolomics data have been deposited to MetaboLights repository with the study identifier MTBLS13406.
